# ‘Leaving the Door Open’: Perspectives on Decision‐Making for Non‐Emergency Diabetes‐Related Amputation

**DOI:** 10.1111/hex.70043

**Published:** 2024-09-26

**Authors:** Emilee Kim Ming Ong, Carolyn Murray, Susan Hillier, Ryan Causby

**Affiliations:** ^1^ Allied Health and Human Performance Unit University of South Australia Adelaide South Australia Australia

**Keywords:** decision‐making, diabetes, diabetes‐related foot ulcers, lower limb amputation, qualitative descriptive study, thematic analysis

## Abstract

**Introduction:**

Having a lower extremity amputation is a life‐changing decision for people living with a diabetes‐related foot ulcer. Although previous research has described both positive and negative lifestyle and function outcomes of diabetes‐related amputations, limited research has been conducted on the decision‐making processes leading up to the amputation. This study aimed to explore the perspectives of persons, healthcare practitioners and experts (including academics and specialists) on decision‐making for people with a diabetes‐related foot ulcer who may require a non‐emergency amputation.

**Methods:**

A qualitative descriptive study using semi‐structured interviews enabled people to share their thought processes when making decisions for amputation. Twenty‐six participants were interviewed, including nine people with a diabetes‐related foot ulcer or amputation, nine health practitioners and eight experts located across five countries. There were 13 female and 13 male participants. Thematic analysis was used for data analysis.

**Results:**

Four themes described the decision‐making considerations for amputation: ‘Balancing the evidence in decision‐making’, ‘Trust, respect and timing of conversations inform decision‐making’, ‘Tailoring decisions for individual circumstance’ and ‘Reaching the tipping point in decisions for the future’. Work commitments, functional and lifestyle impacts of amputation, the presence of support networks and clinical wound features formed the evidence for a decision for amputation.

**Conclusion:**

Understanding quality of life needs ensured that decisions for amputation addressed expectations and lifestyle needs. Living with a diabetes‐related foot ulcer presented daily challenges that pushed people to a tipping point, at which amputation was considered to overcome these hardships and enable them to move on to the next chapter of their life. Further research is required to understand how person‐centred factors can be better incorporated alongside objective clinical assessments in decisions for amputation.

**Patient or Public Contribution:**

People with diabetes‐related foot ulcers, health practitioners and experts shared their perspectives on the decision‐making process for amputation through one‐to‐one interviews. Consideration of the person in the context of their life, environment and personal needs alongside the pathological factors is warranted.

## Practitioner Points

- The decision for amputation incorporates a balance between health concerns, health practitioner assessments and the quality of life expectations and lifestyle needs of the person with DFU.
- Open discussions about living situation and future goals can determine readiness for amputation and initiate consideration of amputation as a possible option.
- ‘Leaving the door open’ for people with DFU to have ongoing conversations about amputation supports people to feel in control of their decisions.

## 1 Introduction

Diabetes is a prominent chronic health condition with one in 10 adults living with diabetes mellitus worldwide [1]. A diabetes‐related foot ulcer (DFU) is a common complication of diabetes, which can increase a person's risk of having a lower extremity amputation (LEA) [2]. Common negative effects of living with a DFU or undergoing an LEA include mobility restrictions imposed by custom medical footwear, financial stressors from costs of wound dressings and loss of employment and emotional distress due to concerns about future independence [3, 4, 5, 6]. The negative impacts of amputation are amplified when people are required to undergo an emergency amputation that occurs suddenly and unexpectedly [3]. Similarly, when not an emergency, the decision can be emotional and multifaceted [3, 4].

Clinical guidelines and wound classification systems are used by health practitioners (HP) to assess a wound and develop an evidence‐based treatment plan for a person with a DFU [7]. However, these guidelines are informed by objective clinical assessments and are usually applied in emergency situations with life‐threatening circumstances. There is a lack of guidance for HP or people with chronic DFU who are considering a non‐emergency amputation for personal and lifestyle reasons. Previous literature describes improvements in quality of life, function and pain management as motivators for a person to consider a non‐emergency amputation [8]. Once there has been time to adjust, the LEA has been reported to increase quality of life by reducing unnecessary long‐term challenges associated with the management of a DFU [3, 9, 10]. Therefore, the choice to continue with conservative wound management or undergo non‐emergency LEA can be challenging and complex for all those involved in considering the decision.

Existing research presents conflicting evidence about the appropriate timing for HPs to initiate conversations about non‐emergency amputation [9, 11]. HPs often do not discuss the possibility of amputation earlier than required to minimise unnecessary fear and distrust, should an amputation not be indicated later [11]. However, Torbjörnsson et al. [9] suggest that non‐emergency amputations should be presented as a treatment option for people with poor wound healing ability. Despite this, the process for discussing non‐emergency amputation with a person with DFU and the involvement of the person and members of the healthcare team in this decision is unclear.

The care and information provided by HPs can impact discussions and decisions for amputation [9, 11, 12]. People who undergo a diabetes‐related LEA often have lower health literacy, which can influence their ability to engage in decision‐making in an informed way [13, 14]. Current processes of decision‐making for non‐emergency amputation incorporate clinical wound features, as well as personal and lifestyle factors as key considerations for amputation [8]. Understanding more about thought processes for decision‐making will support HPs to work collaboratively with people who have DFU when making decisions for amputation.

This research aimed to explore the experiences of persons, HPs and experts in decision‐making in the management of people with a DFU who may require an amputation. This study was guided by the research question, What are the perspectives of people with DFU, HPs and experts about the decision‐making and experience of LEA?

## 2 Methods

This study followed the Consolidated Criteria for Reporting Qualitative Research (COREQ) checklist [15]. Ethical approval for this study was obtained from Southern Adelaide Local Health Network (SALHN) and the University of South Australia Human Research Ethics Committees (SALHN ethics reference number LNR/23/SAC/85).

### 2.1 Study Design

This was a qualitative descriptive study using semi‐structured interviews and thematic analysis to explore and report on the perspectives and experiences of people with DFU, HPs and experts in the decision‐making process for non‐emergency amputation. Although it includes interpretation of the data by researchers, qualitative descriptive research provides a straightforward description of a phenomenon using the participants' language [15, 16, 17].

### 2.2 Sampling and Recruitment

People living with a DFU or amputation who were receiving treatment from a multidisciplinary foot clinic at a public hospital in Adelaide were purposively recruited by the principal researcher. HPs and experts working in multidisciplinary foot clinics or diabetes associations were invited to participate through email correspondence and the researchers' professional networks. A HP was defined as a clinician directly providing DFU treatment to people, such as a podiatrist or nurse, and an expert was involved in a health department or organisation that promotes the management of diabetes foot complications and amputation but may not be directly providing DFU treatment. The eligibility criteria for participants are included in Table 1. Snowball sampling was also undertaken by asking participants to pass on study details to other people who may be interested.

**Table 1:** Eligibility criteria for participants.

Population	Inclusion criteria	Exclusion criteria
People living with a DFU and/or LEA	– Currently receiving treatment from a high‐risk foot service for a DFU – History of LEA due to a DFU within the past three years. This includes people who have undergone a minor or major amputation	– Current or history of ulcer or amputation of other aetiology (non‐diabetes) – Cognitive or communication disorder that would impact the ability to describe experiences in an interview – Limited English proficiency
Health practitioners	– Currently providing DFU treatment in a tertiary care health service – Minimum 1 year of experience managing people who have had a diabetes‐related amputation – This included podiatrists, vascular nurses, vascular or orthopaedic doctors, endocrinologists and orthotists	– Not currently working in a tertiary care health service providing DFU treatment – Limited English proficiency
Experts	– People who are involved in public health departments or non‐government organisations promoting foot health, diabetes care or the management of diabetes‐related foot complications	– No experience in managing or communicating with people who currently have a DFU or history of diabetes‐related LEA – Limited English proficiency

Abbreviations: DFU = diabetes‐related foot ulcer, LEA = lower extremity amputation.

Previous qualitative studies using semi‐structured interviews suggest that sample size is dependent on the research design and aims, with 6–10 participants required for depth [18]. Therefore, the sample size in this study comprised 24 interviews, including eight people with a DFU and/or amputation, eight HPs and eight experts to allow for sufficient data.

### 2.3 Data Collection

Semi‐structured, one‐to‐one interviews were conducted in a quiet room at the hospital or via online video call (Zoom Communications Inc, San Jose, California, United States) for participants located interstate or internationally. All interviews were undertaken by the principal researcher; there was no existing relationship before the interview. The semi‐structured design enabled probing questions to be asked in response to the participants' answers [19]. Two separate interview guides containing similar questions were used; one for HPs and experts, and one for people with DFU (File S1). The interview guides started with general demographic questions to establish rapport and contained open‐ended questions and prompts developed with consideration of the World Health Organisation's International Classification of Functioning, Disability and Health (ICF) framework [20]. Examples of the interview questions that applied to both participant groups include the following:

- What do you think about your involvement in making decisions about the management of DFU or amputation due to DFU?
- Tell me about your thought processes and considerations for deciding about DFU management or LEA due to a DFU.

To check the suitability of questions and allow the interviewer to rehearse their technique, the HP/expert interview guide was piloted with a HP who had relevant clinical and research experience in DFU to provide feedback. Similarly, the interview guide designed for people with DFU was piloted with two people accessing services at a podiatry clinic but did not have an active DFU. All interviews lasted between 30 and 65 min (mean 35 min), were audio recorded and transcribed verbatim by the principal researcher [21]. Field notes were recorded by the principal researcher after each interview to contribute to the analysis [22].

### 2.4 Data Analysis

Data analysis followed a six‐phase reflexive thematic analysis technique described by Braun and Clarke [21]. Microsoft Word (Microsoft 365, Microsoft Corporation, Washington, United States) was used to manage the analysis process. Initially, data from HPs and experts were analysed independently of data from people with DFU but these data sets were integrated to develop the final themes.

Through an inductive process, each interview transcript was read and reread by the principal researcher to immerse themselves in the data, and initial notes were taken on the meanings derived from the text. The principal researcher worked systematically through the transcripts to identify codes, which captured key concepts of interest. Six transcripts were double coded by another member of the research team, which enabled discussion about different interpretations of the data among the research team. Once all data were coded, codes presenting a similar meaning were sorted into categories during a meeting including the whole research team. These categories were then further analysed by the primary researcher and discussed among the research team to arrive at higher order themes. Next, themes and subthemes were reviewed and refined to ensure that they each represented a unique idea. An audit trail of refinements throughout the analysis was used to keep a record of decisions. The final reporting of themes included direct quotations from the participants to ensure the confirmability of the researcher's interpretation [21].

### 2.5 Rigour

Peer debriefing with the research team was conducted throughout data collection and analysis [23]. Confirmability was maintained through an audit trail, and a reflexive journal was used to record field notes, observations and any personal biases [15, 23]. A reflexivity session with the research team was conducted before data collection to document personal experiences and assumptions about DFU management and any possible influences on data interpretation [24]. Demographic details and rich descriptions were used when reporting findings to assist the transferability of findings to other contexts [15].

### 2.6 Author Positioning

The principal researcher is a PhD candidate and practising clinical podiatrist. The co‐authors have a podiatry, physiotherapy and occupational therapy background in clinical practice and research. All authors have experience in conducting qualitative research and have published articles in this area.

## 3 Results

Twenty‐six participants were recruited between September 2023 and January 2024. Demographic information for the participant groups is listed in Tables 2 and 3. There were 13 females and 13 males, located across five countries, with all the people with DFU being from Australia. The HPs and experts were located in Australia (*n* = 9), New Zealand (*n* = 1), Portugal (*n* = 1), United Kingdom (*n* = 5) and the United States (*n* = 1). The mean age of the people with DFU (P) was 63 (SD 3; range 54–80) years, HP was 40 (SD 8; range 28–50) years and 46 (SD 8; range 37–62) years for the experts (E). The mean duration of experience for the HP within their respective fields was 14 years (range 2–30) and 17.5 years for experts (range 3–31). It was the first experience of a DFU for four people, and five had previously had an LEA ranging from a digital amputation to a below‐knee amputation.

**Table 2:** Participant demographic data of people with a DFU and/or amputation.

Participant code	Age	Gender	Employment status	Living arrangements	Duration of ulcer	Amputation history
P01	71	F	Unemployed	Live‐in carer	2 months	Below‐knee amputation
P02	54	M	Unemployed	Housemate	3 years	Hallux amputation and forefoot amputation
P03	62	M	Unemployed	Wife	1 day	Fifth digit amputation
P04	69	M	Retired	Brother	4 years	Below‐knee amputation
P05	59	M	Casual work	Wife	8 months	Nil
P06	63	M	Unemployed	Wife	1 year	Nil
P07	50	M	Unemployed	Alone	6 weeks	Nil
P08	80	M	Retired	Wife	2 years	Hallux amputation
P09	55	M	Carer	Mum	5 years	Nil

Abbreviation: P = person with DFU.

**Table 3:** Participant demographic data of experts and health practitioners.

Participant code	Age	Gender	Country	Occupation	Years of experience
E01	37	M	Australia	Medical scientist	3
E02	42	M	USA	Researcher and podiatric surgeon	10
E03	39	F	Portugal	Podiatrist and researcher	18
E04	40	F	Australia	Podiatrist and researcher	17
E05	47	F	Australia	Podiatry researcher	23
E06	62	F	UK	Diabetologist	23
E07	53	F	UK	Podiatrist and researcher	31
E08	49	M	UK	Vascular surgeon	15
HP01	50	F	Australia	Podiatrist	30
HP02	31	F	Australia	Podiatrist	9
HP03	31	F	UK	Vascular surgeon registrar	5
HP04	39	M	UK	Rehabilitation medicine consultant	7
HP05	28	F	Australia	Prosthetist and orthotist	6
HP06	51	F	Australia	Podiatrist	28
HP07	44	M	Australia	Podiatrist	22
HP08	45	F	New Zealand	Podiatrist	19
HP09	49	F	Australia	Amputee support nurse	2.5

Abbreviations: E = expert, HP = health practitioner.

**Figure 1:**
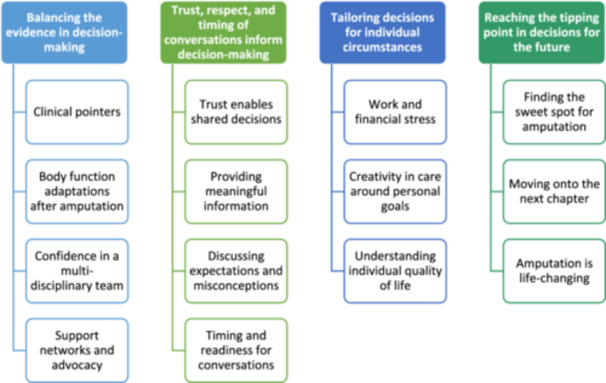
Themes and subthemes.

Four themes (each with subthemes) described the decision‐making considerations for amputation among people, HPs and experts: ‘balancing the evidence in decision‐making’, ‘trust, respect and timing of conversations inform decision‐making’, ‘tailoring decisions for individual circumstance’ and ‘reaching the tipping point in decisions for the future’ (Figure 1).

### 3.1 Theme 1: Balancing the Evidence in Decision‐Making

The decision for amputation involved weighing up the evidence of objective wound assessment features and a HPs clinical expertise. The balancing of evidence included understanding comorbidities, daily activities, previous treatment history and personal perspectives about their physical and psychological well‐being. This goal‐centred, staged approach supported a shared decision for amputation that addressed an individual's preferences alongside evidence‐based recommendations and guidelines.

#### 3.1.1 Clinical Pointers

HPs considered both subjective indicators and objective signs to guide their decisions for amputation. Objectively, type of wound, presence of tissue loss or osteomyelitis were common assessment features. Subjectively, the HP was aware of the preferences and choices of the person with DFU.

As much as there are key clinical pointers that might say a person's not going to heal, the person might be happy to have a palliative wound and not do anything about it.

(HP07)

Surgeons were reluctant to undertake amputation for people experiencing pain (HP04) due to the risks of developing phantom limb pain or neuropathic pain after an amputation; yet pain from the DFU was a considerable concern for P06 who asked to have an amputation that was declined. PO9 expressed frustration about the management of their pain and the options available.

No‐one has an idea about pain management. They've got nothing to say on the subject, other than what not [medications] to take. There's never an answer.

(P09)

The clinical reasoning behind HPs' decisions for amputation was guided by risk classification systems and wound management principles. A ‘limb salvage’ approach to DFU management was inconsistently described by participants. Some HPs endeavoured to save a limb and avoid amputation, whereas others preferred to intervene with amputation sooner as an opportunity for better outcomes. Some HPs reflected that focusing on preserving as much of the limb as possible can lead to subsequent ulcerations that require further amputation in the future.

There was a patient, where in hindsight, we should have had that discussion much sooner and our best intentions of limb salvage probably in that situation were misplaced.

(E07)

Some believed that selecting a suitable level of amputation upfront prevented future ‘salami’ amputations, subsequent ulcerations, delays in healing and repeated exposure to operative and anaesthetic risks. P01 agreed with this sentiment and gave consent for the surgeon to do what was required while she was under anaesthesia.

I just said to the specialist … if you find that it's not going fix the problem, don't come out and tell me that's not going work and I need to go back a second time.

(P01)

Consideration of function at each level of amputation meant one HP (HP04) often recommended a below‐knee amputation, even if the ankle joint was functional, to enable more options for functionality and prosthesis use.

#### 3.1.2 Body Function Adaptations After Amputation

Amputation created biomechanical changes to foot structure, which impacted mobility and engagement in daily activities.

Even a minor amputation changes gait and biomechanics to the foot.

(E01)

Amputation was considered advantageous by some HPs to address major structural deformities in the feet. A person's mobility levels and function before amputation were used by HPs to predict mobility outcomes after an amputation. The risk of subsequent ulceration, pain and deterioration in the opposite limb or the residual limb after an amputation was also considered.

If they have a prosthetic limb, they not only need a healthy residuum, they still need their native contralateral leg to be in a good condition to weight‐bear to help them walk.

(HP04)

#### 3.1.3 Confidence in a Multidisciplinary Team

A multidisciplinary team approach to decision‐making for amputation supported people to feel confident in their decisions. Having the expertise of HPs from various disciplines to work closely together and ‘look at the problem from their angle’ (HP03) boosted confidence from knowing that all aspects of a person's needs were being addressed during decision‐making about amputation.

Decision‐making for amputation is comfortable as we have included other practitioners.

(HP03)

The input of podiatrists was valued with them leading the discussion about amputation in some situations as they had developed rapport and trust through a long‐term relationship with the person.

I don't make any of the decisions, I rely on the podiatrist to make the decisions, what it (DFU) needs, what's best for it.

(P03)

#### 3.1.4 Support Networks and Advocacy

Family and support networks were advocates for care needs, hospital appointments, listening to HPs and providing supported decision‐making for amputation. HPs reflected on how this advocacy was particularly important for people living with cognitive changes who required assistance from their family or next of kin to make an informed decision.

Involve the patient and family in any decisions. We are providing patient‐centred care all the time and explain the complications because we never give any false promises.

(HP09)

People with DFU were grateful for their family's support in decision‐making processes and managing their needs both before and after amputation. Both P05 and P06 commented on their relationships with their wives as being a ‘partnership’ (P06) and that they made decisions together.

She (wife) needed to be involved because if something were to happen and I lost my foot, then she's going to have to be taking care of me until I get back on my foot.

(P05)

### 3.2 Theme 2: Trust, Respect and Timing of Conversations Inform Decision‐Making

Mutual trust and respect between HPs and people with DFU informed decisions for amputation. Reciprocal information sharing involved HPs explaining evidence about amputation and people with DFU sharing information about their treatment goals and expectations.

#### 3.2.1 Trust Enables Shared Decisions

Building rapport and trust supported people to feel in control of their choices. Two people with DFU described having a trusting relationship with their HPs and said they felt comfortable with their expertise and advice. In the absence of trust, people were not likely to attend to their care needs, were not receptive to information and may cling to misconceptions.

If they don't trust you, they're not going to turn up.

(E05)

When there was a consistent HP, people with DFU felt that they were ‘in good hands’ (P08). HPs who listened, respected and responded to a person's needs promoted trust. Both HPs and experts recognised that respecting and acknowledging an individual's wishes gave time for decision‐making without the individual feeling forced.

It's never my decision. It's always their decision. All I can do is help them come to what I think might be the right decision*.*

(E04)

They take onboard my thoughts and how I feel and everything*.*

(P01)

Despite HPs respecting the personal decisions of people with DFU, shared decision‐making was not universally experienced, with several people with DFU not feeling involved in the decision‐making process for their amputation creating frustration and distrust.

I don't get any say, I just follow the instructions (for amputation).

(P06)

#### 3.2.2 Providing Meaningful Information

Understanding the evidence and risks for amputation through the provision of meaningful information helped people with DFU to recognise the implications of their individual circumstances, and therefore come to a decision. Expert E07 would work hard to give both the person with the DFU and their surgeon the same information so they were ‘both on the same page’. This involved providing research evidence and recommendations using suitable language, which could be understood by the person. There was strong advocacy for a collaborative approach to decision‐making that included ‘open discussion with the healthcare services that are all collaborating with this client about levels of amputation’ (HP05).

Patients who are well informed and motivated, who have felt they have been well supported in their decision‐making tend to do better afterwards*.*

(HP04)

Despite experts and HPs recognising the need to inform people about amputation, many people with DFU felt they had insufficient information for decision‐making and wanted more information about the amputation process.

It's all the unknowns, it plays with your head.

(P04)

#### 3.2.3 Discussing Expectations and Misconceptions

Discussing a person's expectations about the amputation process, prognosis and eligibility for prosthesis use gave an opportunity to clarify questions, debunked misconceptions, established trust and established a mutual understanding of expectations. HPs described their role in communicating and clarifying the expectations and risks of amputation while checking that this information was understood by the person with DFU so there was no uncertainty in their decision.

It's weighing up the risks and balances with the patient and understanding why they want the surgery and what they think will happen*.*

(E06)

Despite the ambitions of HPs in sharing information about amputation and the reasoning behind decision‐making, it was not always provided as expected.

They probably should have explained it (amputation) a bit more. What was going on, why they were going to take my toes off versus the foot.

(P04)

There was a common misconception among people with DFU that amputation would help them overcome all their chronic foot complications, and there was sometimes a lack of awareness of the extensive follow‐up care required after their amputation. Checking for misunderstandings and using terminology suitable to the person's health literacy was recommended. Clarifying questions that a person with DFU had about the amputation process appeased their fears about amputation and uncertainty for their future.

Nobody is escaping the statistics (post‐amputation mortality rate)*.*

(E02)

#### 3.2.4 Timing and Readiness for Conversations

There was limited consistency in practice around the timing to have conversations about amputation. Some HPs tried to avoid discussing amputation prematurely and would not bring up amputation ‘lightly’ (E02) unless they believed it was a probability. In contrast, others believed they ‘should not shy away from those conversations’ (HP03) and needed to raise amputation with people with DFU early because they ‘fear[ed] amputation more than death’ (HP07).

When I got the staph infection, they said 99.9% chance you'll be coming out of surgery just below the knee. I was prepared, I was all happy for it*.*

(P02)

Bringing amputation up early is reassuring to the patient because it allows them to have that open discussion, because else it tends to be the elephant in the room.

(E07)

Four people with DFU recalled their HP mentioning amputation early and felt prepared for it due to these early discussions. Others tried to ‘hold off’ on amputation, as they were hesitant to undergo a permanent procedure if there was a chance that their DFU could heal with conservative care.

Several HPs felt frustrated when decisions did not reflect their professional advice and evidence‐based recommendations; however, they recognised the importance of supporting and enabling people to come to their decisions in their own time.

It has to be about informing them, supporting them, even if it's not the choice we would personally make for us and always leaving the door open for the patient (sic) making a different choice.

(E05)

Having a trusted HP ‘plant the seed’ (HP02) of an amputation conversation early and provide the justification for amputation gave rationale behind the recommendation and offered reassurance. This softened the shock of the news about amputation so that conversations about amputation were not ‘out of the blue’ (E05).

### 3.3 Theme 3: Tailoring Decisions for Individual Circumstance

Amputation decisions were tailored around a mutual understanding of a person's living circumstances, work commitments and quality of life needs. Key aspects of lifestyle requirements that fed into decision‐making about amputation were financial security, the goals of the person with DFU and a focus on improving their quality of life.

#### 3.3.1 Work and Financial Stress

People who worked labour‐intensive jobs were self‐employed or needed to drive as part of their work struggled to adapt their work demands or take time off work for wound healing; ‘I'm casual. If I don't work, I don't get paid’ (P05). For these people, a ‘difficult circular situation arose’ (E01) whereby they couldn't rest, so their wound worsened, which meant they needed more treatment that also interfered with their work. In some cases, there were stories of an amputation being a more efficient solution to overcome the challenges associated with offloading a DFU while working. Conversely, conservative treatment was more feasible for people working in sedentary roles because they were able to tolerate a greater extent of offloading, thus delaying any decision for amputation.

After an amputation, some people with DFU remained unable to work and had no financial support due to the age restrictions for disability funding.

I have my own debts, car payments … I had to sell my car, bring my footprint to nothing. All I do now is eat. That's my only footprint*.*

(P06, no pun intended)

HPs considered the financial status of the people with DFU and suggested ‘that eventually ministerial funding will change’ (HP08) to provide more support. They explained that financial status is generally not considered in decision‐making for amputation in Australia, which is a different system in the United States, where insurance status can influence decisions.

#### 3.3.2 Creativity in Care Around Personal Goals

The decision for amputation required a collaborative and creative approach that incorporated a person's goals of care to ‘jigsaw all the pieces of the puzzle together’ (E04) and supported a personalised decision for amputation. For example, wound healing was not the goal for some people who were focused on reducing their hospital visits or engaging in meaningful activities such as going to the beach, which was inhibited by conservative wound care.

If it's patient‐centred and it's always being driven by their goals and priorities, you never run astray because it's being directed by their needs.

(E05)

#### 3.3.3 Understanding Individual Quality of Life

Understanding how each person defined their quality of life enabled decisions that recognised what was important to them.

I'm thinking about quality of life and how viable the foot is, and how the people are feeling, and how their families are feeling.

(HP08)

HP07 noted that ‘quality of life can mean a whole range of things’ such as work, participating in activities of daily living or removing the ‘medical burden’ of a DFU. For people living with DFU near end of life or in palliative care, conservative wound care was considered more appropriate to ensure they remained as comfortable as possible.

Some people die with the diseased limb on, because that's genuinely the best for their circumstances because they cannot get around without a limb*.*

(E05)

According to E05, a person with cognitive changes or dementia may not be suitable for an amputation or prosthesis after an amputation, as they may not remember having the limb removed, how to use a prosthesis or successfully engage in rehabilitation. In contrast, for someone who was systemically well, an amputation may be recommended to optimise their quality of life.

### 3.4 Theme 4: Reaching the Tipping Point in Decisions for the Future

This theme describes the point at which people living with a chronic DFU decided to move beyond their current pattern of care and management. This tipping point was prompted by a combination of health issues, running out of options for their DFU and reaching a point of readiness to consider amputation for future quality of life.

#### 3.4.1 Finding the Sweet Spot for Amputation

The ‘sweet spot’ for amputation largely centred around the person still being healthy enough to make active decisions for their care, adequately prepare for an amputation, heal and rehabilitate well.

There can be a sweet‐spot for amputating a leg. It's ensuring that it's the right time for them in their overall health to be able to rehab afterwards.

(E05)

Deciding to amputate during this ‘sweet spot’ may mean that ‘rehabilitation outlooks are a bit better’ (HP03). People with DFU may reach a point where they can see the value in early intervention to prevent further complications, deterioration and repeated hospital admissions that were stressful.

In and out (of the hospital). It's too much trauma, too much stress for me to handle.

(P01)

The age and condition of the person were part of the reasoning in deciding the ‘sweet spot’. Younger people who were relatively active could ‘have one surgery and get great quality residuum’ leading to a ‘good prosthesis’ (HP05) and recovery. Some people who persisted with managing their DFU through conservative treatment left it too long to decide, and the ‘sweet spot’ for amputation was missed. They were running out of options for conservative treatments, and the decision was becoming inevitable.

They've said I'm going to lose my leg at some stage. It's not if, but it's when.

(P02)

The decision sometimes has already been made because they've tried everything else.

(HP04)

Delaying the decision precipitated the likelihood of an emergency amputation, which was stressful due to being unprepared and support networks not being present at short notice. Missing the window of opportunity meant that people with DFU lost condition and became frailer.

He would have done very well with a high‐level amputation a long time ago … he's basically non‐weightbearing. He's completely deconditioned and when the time comes … he's not going to be able to rehab.

(E04)

#### 3.4.2 Moving on to the Next Chapter

Commitments to regular DFU management introduced considerable lifestyle interruptions for many people, which were described as stuck ‘in a loop’ (P09) and led to continual worry about their DFU deteriorating.

Some people will try and preserve their foot at all costs and other people will be quite ready to consider amputation if it means that they can get on with their life.

(E08)

This stasis and worry became so disruptive to daily life that this often became the tipping point with people opting for amputation to break the cycle they were in and move on to the next chapter of their life.

What's important to them in terms of what they want to do with their days? What is it that having a foot ulcer is preventing them doing, that an amputation might enable them to do?

(E07)

I said if I present one more time, I'm going to bring my saw with a brand new blade. If you don't cut it off in the first 24 hours, I'll cut it off in my bed and you can clean up the mess*.*

(P02)

#### 3.4.3 Amputation Is Life‐Changing

When the tipping point was reached, amputation introduced permanent changes to a person's life, including their ability to work and participate in activities of daily living. One person with DFU (P01) was challenged by the lack of accessibility in their environment and struggled to venture out to new places in their wheelchair after the amputation. Amputation was a major decision with emotional and physical implications.

It's the most confronting medical procedure that you can have, literally having a part of your body removed.

(E01)

Reassurance about the support available, milestones of healing, prosthetic rehabilitation and how they will re‐enter the community safely after amputation was helpful. HPs provided these timelines with the caveat that each person's healing capacity differed and acknowledged the difficulties of accurately predicting healing and rehabilitation after an amputation.

## 4 Discussion

This study aimed to explore the experiences of decision‐making for non‐emergency LEA due to DFU. The decision‐making process for amputation involved a combination of clinical DFU features and the individual's circumstances. Decisions aimed to achieve a person‐centred approach for non‐emergency LEA.

The contribution of evidence such as wound characteristic assessment, use of clinical guidelines and functional assessment towards HPs' clinical reasoning for LEA has been previously reported in the literature [25, 26]. However, this study highlighted the need for balance between these assessment features and person‐centred factors such as emotional state, psychological well‐being and support networks; with some HPs and experts placing a greater emphasis on the personal factors when making decisions for non‐emergency amputation. Sometimes this need for balance created reasoning tensions for HPs when trying to compromise between a clinical evidence‐based approach using their professional expertise and a person's individual circumstances. This multifactorial approach to evidence‐informed decisions is consistent with the pillars of evidence‐based practice, which incorporate research evidence, clinical expertise, patient values and circumstances and contextual evidence [27]. However, this study highlighted the inner conflict that HPs and experts experience when balancing the priorities of the four pillars including a person's health, current living circumstances and future when making decisions about amputation. This demonstrates a need for further training for HPs about the use of evidence‐based approaches that incorporate a person's clinical presentation, personal circumstances (including health literacy) and their own clinical expertise and work experience.

Understanding how a person defines their quality of life and what is important to them in their daily life determined whether amputation was appropriate for the individual's circumstances. Quality of life is also a key consideration in decisions within end‐of‐life care [28, 29]. However, a tailored approach was not always experienced by people with DFU in this study who reported decisions being made on their behalf without consideration of their preferences. Nevertheless, this study highlights the positive amputation experiences and outcomes that can result from a collaborative approach to decision‐making.

The findings demonstrated that reciprocal sharing of information, goals and evidence between the person with DFU and their clinical team ensured an ongoing partnership between all parties involved in the amputation decision [30]. These findings build on previous research on the Patient Activation Measure (PAM) that assesses a person's knowledge, skills and confidence within their ability to manage their own health [31]. People with DFU in this study demonstrated varied levels of knowledge and confidence to lead the decision for amputation. This is consistent with the findings of Kearns et al. [32] who highlighted goal setting and provision of health information as common strategies to implement PAM to achieve tailored person‐centred care [31]. It is possible that the literature on collaborative approaches and the empowerment of individuals in the decision‐making process has not yet fully made its way into this field. In other fields of healthcare, such as decision‐making for total knee replacement in knee osteoarthritis, research has shown that people want additional information about their surgery to manage their expectations [33]. Our research concurs that there is a place for collaboration and empowerment for supporting HPs, experts and people with DFU with the high‐stake decision of non‐emergency LEA.

A person's ability to trust their HPs is important for shared decision‐making. In the presence of trust, people with DFU could engage in honest conversations with their HPs about their expectations of amputation and the goals they hoped to achieve with an amputation. The importance of trust in person–practitioner relationships for shared decision‐making identified is consistent with previous studies exploring the role of trust in clinical decision‐making [33].

This study identified different expectations and desires for participation in the decisions for LEA among people with DFU. People with DFU described their thought processes as they reached a pivotal tipping point during their conservative wound management, which led to their decision to undergo LEA and move on to the next chapter of their life. People with DFU experienced internal conflicts around where this point was and were often deciding back and forth between continuing with conservative wound care or proceeding with amputation. Determining a person with DFU's readiness to undergo amputation and timing to initiate these conversations was difficult for HPs. This highlights the importance of a personalised approach to amputation decisions that empowers people with DFU to participate at a level comfortable to them, depending on their readiness to engage. Some people with DFU felt confident to make the decision about amputation on their own, using the information provided by their HP, whereas others preferred a shared decision between the HP, family members and themselves. Moreover, it is also important to understand that not every person is ready to engage in shared decision‐making, and individuals may require personalised support during this process [34]. The data showed the need to ‘leave the door’ open to support people with DFU to change their level of participation and engagement in the decision‐making process depending on their readiness, including giving the opportunity for people who had initially declined amputation, to change their mind without judgement.

## 5 Limitations and Further Research

All people with a DFU were recruited from a metropolitan public hospital in South Australia. Therefore, their experiences of amputation may differ from the perspectives of people living in rural or remote areas and across different states and countries, highlighting the need for further research across different geographical locations where escalation pathways for amputations and resources differ. Most people with DFU in this study were male, which is consistent with the general population of people with DFU who have amputation. However, further exploration of the considerations of people identifying with other sexes could provide a different insight into the thought processes for amputation. The important role of family members in the decision‐making was raised by participants, but because this study sample did not include family members, spouses or carers, this is recommended for future research to provide an additional perspective on the role of support people in the decisions for LEA. Moreover, including Indigenous people in a future study would support more diverse perspectives on decision‐making for amputation, particularly as there can be a high incidence of DFU in this population [35]. Some health disciplines within the multidisciplinary teams involved in DFU management were not involved in this study. Most of the HPs and experts involved in the study were from developed countries, demonstrating the need for further research to explore the decision‐making processes across all members of the multidisciplinary team and a wider variety of countries. The findings from this study will be presented to people with DFU, HPs and experts to gain their perspectives on how these findings can be incorporated into a framework and implemented into clinical practice to support their decision‐making for amputation.

## 6 Conclusion

A person's lifestyle, work commitments and quality of life needs were key considerations when making decisions for non‐emergency amputation. Many people with DFU reached a tipping point with their DFU, where an amputation was considered to overcome the daily challenges of DFU management. The sweet spot for amputation was unique to the individual's circumstances but when identified, supported positive outcomes and quality of life postamputation. There is a need for further research to understand how these person‐centred factors can be balanced alongside clinical assessment due to the complex nature of amputation decision‐making for HPs, people with DFU and their families.

## Author Contributions

**Emilee Kim Ming Ong:** conceptualisation, investigation, writing–original draft, methodology, writing–review and editing, formal analysis, project administration, visualisation. **Carolyn Murray:** conceptualisation, writing–review and editing, formal analysis, supervision. **Susan Hillier:** conceptualisation, writing–review and editing, formal analysis, supervision. **Ryan Causby:** conceptualisation, writing–review and editing, formal analysis, supervision.

## Ethics Statement

Ethics approval was obtained from Southern Adelaide Local Health Network (SALHN) and the University of South Australia Human Research Ethics Committees. All participants provided written informed consent to participate in this study.

## Consent

All patients were informed that participation was voluntary and provided written informed consent before interview. Patients were reminded that participation in this study or withdrawing at any time would not affect their regular care, nor their relationship with staff of the university or health service.

## Conflicts of Interest

The authors declare no conflicts of interest.

## Supporting information

Supporting information.

## Data Availability

Transcripts will not be made available to maintain the confidentiality of all participants.
