# Supplementary material for: ‘Leaving the Door Open’: Perspectives on Decision‐Making for Non‐Emergency Diabetes‐Related Amputation
Source: Health Expect. 2024 Sep 26;27(5):e70043. doi: 10.1111/hex.70043 (PMC11424879; doi:10.1111/hex.70043)
Supplement: Supplementary file 1 — Supporting information. [file HEX-27-e70043-s001.docx]

# Supplementary file 1. Interview guides

## People with diabetes-related foot ulcer interviews

Interview questions

Today, I am really interested in hearing about your decision-making considerations about managing a diabetes-related foot ulcer and elective amputation. But first, I’d like to understand a little bit more about who you are…

**Demographic data for people with diabetes-related foot ulcer**

Self

Please tell me a bit about yourself:

- What is your age and the gender you identify with?
- Where and when were you born and raised?
- Where are you living now? What is your postcode there?
- Are you living with a partner, someone else or living alone?
- Are you working at present? Can you describe your occupation/previous occupation?

History of foot complications

Please tell me a bit about your ulcer and/or amputation:

- Have you had a previous ulcer or is this your first foot ulcer?
- How long have you had this ulcer for?
- How long have you been attending this podiatry clinic for ulcer treatment?
- Have you had a previous lower limb amputation due to diabetes? If so, what type of amputation/s have you had and when did you have the amputation?

**Amputation decision-making**

Current ulcer management

- Tell me about the management of your ulcer. How do you feel the current treatment of your ulcer is going? Is there anything that should be being done different?
- In what ways did your need to work influence your decisions about your foot care?

Decision-making for management

- Tell me about your thought processes and considerations when making decisions about your ulcer management.
- When making these decisions, how was amputation part of your considerations.
- What do you think about your involvement in making decisions about the management of your ulcer and elective foot amputation?
- Is there any information that you wish you had known earlier or are still wanting to know more about?

Amputation

- When an amputation was first discussed, how did you feel?
- Can you describe any unexpected experiences or feelings now that you have had an amputation?

Probes to use with each story

- How did that make you feel? What helped you to manage those feelings?
- How did you make that decision (family, friends, doctor, podiatrist, internet) or what helped you to make that decision?

End

- Is there anything else you would like to share with me about your decision-making processes about ulcer or amputation management?
- Have we missed anything you think is important?
- Is there anything you would like to ask me?

General probes

- Please tell me more about that.
- Then what happened?
- Can you give me an example of what you mean?
- How does that make you feel?
- Please tell me more about that time.
- How has that changed over time?
- Is there anything else you want to add?
- I really want to understand what it is like for you.

## Health practitioner/expert interviews

Interview questions

Today, I am really interested in hearing about your decision-making considerations about managing a diabetes-related foot ulcer and elective amputation. But first, I’d like to understand a little bit more about who you are…

**Demographic data for health practitioners and experts**

Self

Please tell me a bit about yourself:

- What is your age and the gender you identify with?
- What is your occupation? How many years have you been working in this field?
- What is your current role and how many years have you been working in your current position?

**Amputation decision-making**

Current ulcer management

- What influences how you manage a diabetes-related foot ulcer? How does this differ between different patients?
- To what extent is a patient’s need to work considered in your decision-making for their management?

Decision-making for management

- Tell me about your thought processes and considerations for deciding about ulcer management and foot amputation for a patient.
- What do you think about your involvement as a practitioner in making decisions about the management of a patient’s ulcer and foot amputation?

Amputation

- Tell me about what factors you consider for a patient that may benefit from an elective amputation.
- Can you describe a certain experience where you would have done something different in your management of a patient now knowing their outcome.

Probes to use with each story

- How did that make you feel? What helped you to manage those feelings?
- How did you make that decision (family, friends, doctor, podiatrist, internet) or what helped you to make that decision?

End

- Is there anything else you would like to share with me about your decision-making processes about ulcer or amputation management?
- Have we missed anything you think is important?
- Is there anything you would like to ask me?

General probes

- Please tell me more about that.
- Then what happened?
- Can you give me an example of what you mean?
- How does that make you feel?
- Please tell me more about that time.
- How has that changed over time?
- Is there anything else you want to add?
- I really want to understand what it is like for you.
